# Endocyclophotocoagulation Combined with Phacoemulsification in Glaucoma Treatment: Five-Year Results

**DOI:** 10.3390/biomedicines12010186

**Published:** 2024-01-15

**Authors:** Bartłomiej Bolek, Adam Wylęgała, Małgorzata Rebkowska-Juraszek, Edward Wylęgała

**Affiliations:** 1 Chair and Clinical Department of Ophthalmology, School of Medicine in Zabrze, Medical University of Silesia in Katowice, 40-760 Katowice, Poland; 2 Clinical Department of Ophthalmology, District Railway Hospital, 40-760 Katowice, Poland; 3 Health Promotion and Obesity Management, Pathophysiology Department, Medical University of Silesia in Katowice, 40-752 Katowice, Poland

**Keywords:** cyclodestruction, glaucoma, endocyclophotocoagulation, transscleral cyclophotocoagulation, cataract

## Abstract

Background: this study aimed to assess the effectiveness and safety of phaco-endocyclophotocoagulation (phaco-ECP) in patients with glaucoma over five consecutive years. Methods: Thirty-eight patients (38 eyes) with primary and secondary glaucoma were enrolled to undergo phaco-ECP (Endo Optiks URAM E2, Beaver-Visitec International, Waltham, MA, USA). The primary outcome measures were intraocular pressure (IOP) reduction, success rates, glaucoma medication use, and visual acuity after phaco-ECP. An IOP reduction of 20% compared to the baseline value without re-intervention was considered a successful treatment. Complete success was defined as a cessation of antiglaucoma medications. Secondary outcome measures included intraoperative and postoperative complications. Measurements were performed preoperatively and in the first week and 1, 3, 6, 12, 18, 24, 30, 36, 42, 48, 54, and 60 months postoperatively. Results: The mean ± SD values of IOP preoperatively, at 12, 24, 36, 48, and 60 months postoperatively were 22.6 ± 6.7 mmHg, 15.9 ± 3.9 mmHg (*p* < 0.001), 15.9 ± 2.9 mmHg (*p* < 0.001), 15.6 ± 2.7 mmHg (*p* < 0.001), 15.5 ± 3.8 mmHg (*p* < 0.001), and 15.2 ± 2.6 mmHg (*p* < 0.001), respectively. The mean IOP at the last follow-up was reduced by 32.7%. The decrease in the number of antiglaucoma medications was statistically significant at each follow-up visit compared to the baseline. The qualified success rate was 40.6%. All patients at the 60-month follow-up visit required the use of antiglaucoma medications—none of the patients achieved complete success. During the follow-up period, nine patients (28.3%) that required retreatment due to nonachievement of the target IOP were considered failures. Six patients (15.8%) were lost from the follow-up. A total of 23 patients were evaluated 60 months after their phaco-ECP. Complications directly associated with the procedure, such as corneal edema (25.6%), IOP spikes (20.5%), IOL dislocation (2.6%), and uveitis (12.8%), were observed in our patients. Hypotony was not observed in any of our patients. Conclusions: The phaco-ECP procedure was effective, well-tolerated, and safe for reducing IOP in glaucoma patients with cataracts over a long-term follow-up. Randomized, larger-scale studies are required to validate the results obtained.

## 1. Introduction

Cyclodestructive techniques are used to treat moderate and severe manifestations of glaucoma [1,2,3]. These methods induce a decrease in intraocular pressure (IOP) by attenuating the production of aqueous humor through partial damage to the non-pigmented epithelium of the ciliary body. In comparison to the commonly used transscleral diode laser cyclodestruction (TSCPC), endocyclophotocoagulation (ECP) for glaucoma treatment allows for direct visualization of the ciliary processes, precise laser application, and has demonstrated superior safety and selectivity over TSCPC [4,5,6]. More recently, to enhance the safety profile of existing cyclophotocoagulation methods, two innovative approaches have been introduced: micropulse cyclophotocoagulation and ultrasound cycloplasty. In the former, each laser pulse is divided into extremely short-duration phases, thereby minimizing heat accumulation and reducing the disruption of the non-pigmented epithelium and adjacent tissues [2,7,8,9,10]. In the latter, high-intensity focused ultrasound technology is utilized, and energy is delivered via a specially designed probe. This technique enables precise focusing of ultrasound energy at the desired depth, minimizing uncontrolled absorption and thereby reducing damage to adjacent tissues [11,12,13]. In juxtaposition to the transscleral methodology, ECP enables the precise titration of diode laser therapy targeting the ciliary body through direct visualization via endoscopy. This ability to directly observe the target tissue represents the foremost benefit of this method. Nonetheless, it is an invasive procedure typically indicated for patients with glaucoma who are concurrently undergoing cataract surgery [14,15].

To date, only one study with a follow-up period exceeding 36 months has assessed the effectiveness of endoscopic cyclophotocoagulation (ECP) or its combination with phacoemulsification (phaco-ECP). This study aims to assess the effectiveness and safety of the phaco-ECP treatment in glaucoma patients over a consecutive 60-month follow-up period. The primary outcome measures included post-procedure intraocular pressure (IOP) reduction, success rates, glaucoma medication usage, and visual acuity. Secondary outcome measures encompassed any procedure-related complications or postoperative adverse events. The extended period between surgery and the last follow-up visit enables a more precise determination of the effectiveness of the procedure and the need for reintervention, which is crucial in glaucoma management for the preservation of visual acuity.

## 2. Materials and Methods

This was a non-randomized, prospective, single-arm, single-center, follow-up clinical study. It received approval from the institutional review board of the Medical University of Silesia (KNW-0022-KB1-131-16) and followed the tenets of the Declaration of Helsinki. Patients were recruited between November 2016 and September 2018. All enrolled patients provided written informed consent prior to participating in the study.

Inclusion criteria for the study encompassed adult patients (≥18 years) with uncontrolled glaucoma (IOP > 21 mmHg, despite the maximum tolerated doses of antiglaucoma medications) or intolerance to glaucoma medications despite well-controlled IOP. Exclusion criteria included pregnant women, ocular and non-ocular disorders that can significantly affect the visual field other than glaucoma or cataract, and patients less than 18 years of age. Comprehensive ophthalmic examinations were conducted, which involved measurements of IOP using the standard Goldmann applanation tonometer (GAT), an assessment of the number of antiglaucoma medications, a and determination of best-corrected logMAR visual acuity. These examinations were performed preoperatively and at 1 day, 1 week, 1, 3, 6, 12, 18, 24, 30, 36, 42, 48, 54, and 60 months postoperatively. The IOP measurements adhered to the guidelines set forth by the World Glaucoma Association for the design and reporting of glaucoma surgical trials [16]. Visual field tests were performed using a Humphrey Visual Field Analyzer III (Carl Zeiss Meditec, Dublin, CA, USA) set for a full threshold 24-2 testing strategy preoperatively and at 12, 24, 36, 48, and 60 months postoperatively. To measure VF loss the mean deviation (MD) parameter was used. Glaucoma stages of patients in the study group were based on the MD (early glaucomatous loss—MD ≤ 6 dB; moderate glaucomatous loss—6 dB > MD ≤ 12 dB; advanced glaucomatous loss—MD > 12 dB) [17]. The study considered a treatment successful (qualified success) if there was a 20% reduction in IOP and the IOP remained below 21 mmHg during the 60-month period of follow-up visits compared to the baseline value without requiring additional surgical intervention [16]. Complete success was defined as a cessation of antiglaucoma medications. Failure was defined as the IOP not being reduced by at least 20% from the baseline and above 21 mmHg (over two consecutive visits), and/or the necessity for further glaucoma surgical intervention. In cases where IOP was not sufficiently lowered during follow-up and the number of antiglaucoma medications was reduced compared to the baseline, additional medications were added. If needed, further glaucoma surgery was performed based on the patient’s clinical conditions.

Standard phacoemulsification surgery was performed, with intraocular lens implantation in the capsular bag. An ECP (Endo Optiks URAM E2, Beaver-Visitec International, Waltham, MA, USA) probe was carefully introduced into the sulcus, and diode laser treatment was applied between 270 and 360 degrees, continuing until whitening and shrinkage of the ciliary processes were observed. The power was initially set at 250 mW and was then adjusted based on the observed effect.

All surgical procedures were executed by one unchanging surgeon (E.W.). Postoperative management involved a reassessment of intraocular pressure (IOP), with adjustments made to the preoperative antiglaucoma medication regimen based on these readings. Subsequent to surgery, patients were administered topical ofloxacin (five times a day for two weeks) and dexamethasone (five times a day for two weeks, followed by three times a day for two weeks). Statistical analyses were carried out using Statistica Software version 13 (TIBCO Software Inc., Palo Alto, CA, USA). Group comparisons for specific parameters were conducted using either the Wilcoxon signed-rank test or a paired t-test, contingent upon the data distribution. Kaplan–Meier survival curves were employed to evaluate the qualified and complete success of the treatment over time. The results of IOP are visually represented through a scatter plot, illustrating preoperative IOP on the *x*-axis versus 60-month postoperative IOP on the *y*-axis. A *p*-value of ≤0.05 was deemed statistically significant.

## 3. Results

Thirty-eight patients (38 eyes) diagnosed with primary and secondary glaucoma were included in this study of phaco-ECP. Detailed patient characteristics can be found in Table 1. The glaucoma stages of the patients enrolled in the study, based on their VF, are presented in Table 2. Preoperatively, sixteen of our patients had early glaucomatous loss, thirteen of our patients had moderate glaucomatous loss, and 9 of our patients had advanced glaucomatous loss. Throughout the follow-up period, nine eyes (28.3%) required re-treatment due to not achieving the target IOP and were categorized as failures. Among these, three eyes (7.9%) underwent TSCPC surgery approximately 23 months later, two patients (5.3%) received UCP treatment around 30 months post procedure, one patient (2.6%) underwent trabeculectomy 18 months after the initial intervention, and microshunt implantation was performed in two patients (5.3%) approximately 42 months later. Additionally, one patient (2.6%) had non-penetrating deep sclerectomy 24 months following the phaco-ECP. Six patients (15.8%) were lost to the follow-up during the study period. Of these, three patients died from causes unrelated to glaucoma, while three patients discontinued their participation in the study visits due to reasons of a medical or non-medical nature that were unrelated to glaucoma. As a result, a total of 23 patients were included in the evaluation at the 60-month follow-up following their phaco-ECP.

**Figure 1:**
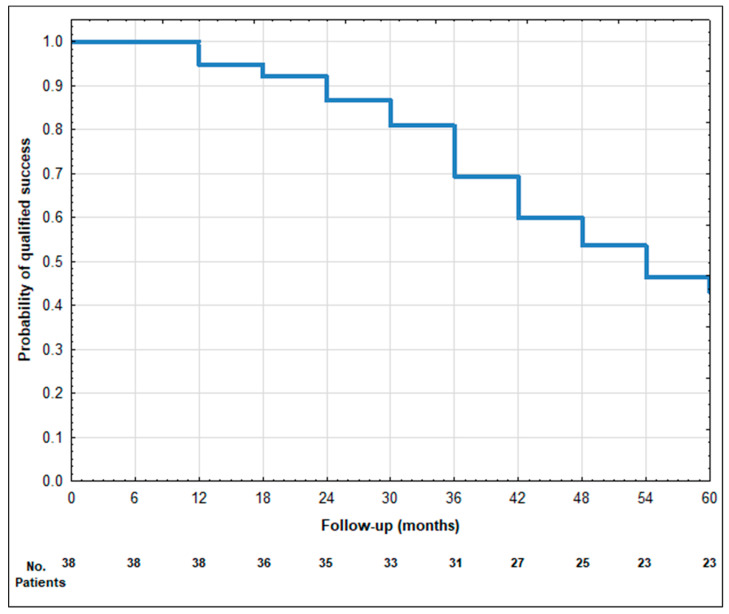
Kaplan–Meier survival curve of qualified success after phaco-ECP—60-month follow-up.

**Figure 2:**
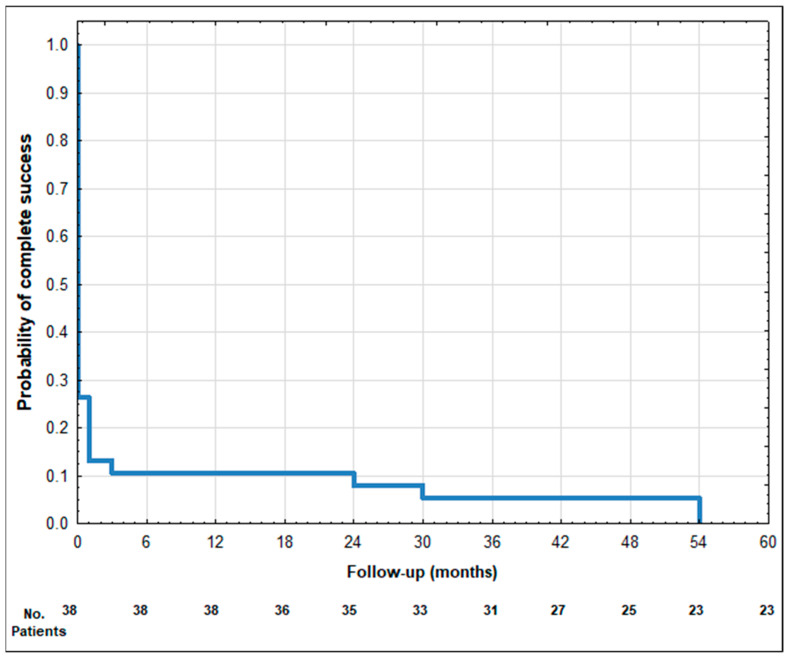
Kaplan–Meier survival curve of complete success after phaco-ECP—60-month follow-up.

**Figure 3:**
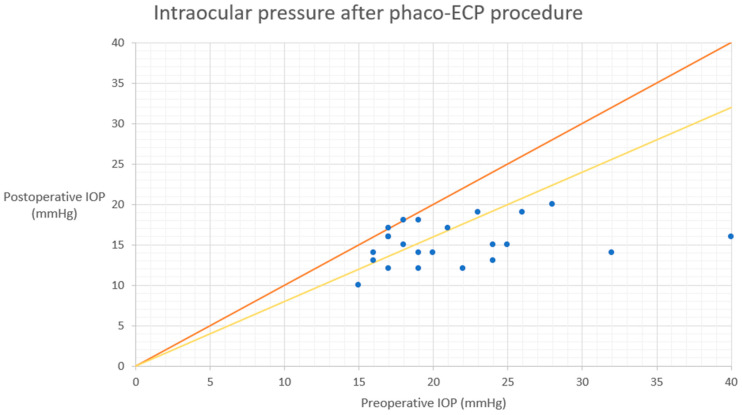
Scatter plot of preoperative IOP (*x*-axis) versus 60-month postoperative IOP (*y*-axis). Each point represents one eye showing the preoperative IOP and the postoperative IOP. Orange line indicates no change. Yellow diagonal line represents 20% IOP reduction. phaco-ECP—phaco-endocyclophotocoagulation, IOP—intraocular pressure.

The mean ± SD values of IOP preoperatively at 1 day, 1 week, 1, 3, 6, 12, 18, 24, 30, 36, 42, 48, 54, and 60 months postoperatively are presented in Table 3. A statistically significant reduction in IOP was observed at each follow-up timepoint compared to the baseline. The mean IOP at the last follow-up showed a reduction of 32.7% (Table 3). The qualified success rate was 40.6% (13 out of 32 eyes). Among the patients who did not achieve qualified success, nine eyes required additional surgical interventions. Furthermore, ten patients did not meet the minimum IOP reduction success criterion. At the 60-month follow-up, all patients still required antiglaucoma medications, and none achieved complete success. Visual representations of the data are provided as the Kaplan–Meier survival curves (Figure 1 and Figure 2) and a scatter plot (Figure 3). It is important to note that patients lost to the follow-up were excluded from the analysis of success rates.

The mean ± SD values of the number of antiglaucoma medications taken preoperatively and 1 day, 1 week, 1, 3, 6, 12, 18, 24, 30, 36, 42, 48, 54, and 60 months postoperatively are presented in Table 3. A statistically significant reduction in the number of antiglaucoma medications was observed at each follow-up timepoint compared to the baseline. Before undergoing phaco-ECP, three patients required systemic carbonic anhydrase administration. During the 60-month follow-up, thirteen eyes maintained the same number of antiglaucoma medications as at baseline. Conversely, other patients showed a reduction in their number of antiglaucoma medications at the last follow-up compared to baseline. None of the patients experienced an increase in the number of antiglaucoma medications compared to their preoperative regimen during the last visit. In a few cases, systemic carbonic anhydrase was added during follow-up just before surgical re-intervention to lower intraocular pressure, and these cases were considered treatment failures. None of the eyes required systemic carbonic anhydrase at the 60-month follow-up.

The best-corrected logMAR visual acuities ± SD values preoperatively and 1 day, 1 week, 1, 3, 6, 12, 18, 24, 30, 36, 42, 48, 54, and 60 months postoperatively are presented in Table 3. There were statistically differences in this parameter at each significant follow-up timepoint compared to the baseline. 

The VF mean deviation ± SD values preoperatively and 12, 24, 36, 48, and 60 months postoperatively were −9.07 ± 7.50 dB, −9.59 ± 10.26 dB (*p* = 0.215), −9.44 ± 8.71 dB (*p* = 0.255), −10.26 ± 9.41 dB (*p* = 0.483), −9.21 ± 8.01 dB (*p* = 0.937), and −9.46 ± 10.03 dB (*p* = 0.191), respectively.

Table 4 lists the postoperative complications encountered during the study. Corneal edema was observed in ten patients (26.3%), which resolved over time without corneal decompensation. An IOP spike occurred in eight patients (21.1%). In one patient (2.6%), there was an IOL dislocation (subluxation) 36 months post-procedure. Three patients (7.9%) developed macular edema, while four patients (10.5%) experienced the development of an epiretinal membrane approximately 46 months after the phaco-ECP procedure. One patient (2.6%) encountered a full-thickness macular hole 12 months after the initial procedure. Anterior uveitis was observed in five patients (13.2%) immediately after the procedure. Five patients (13.2%) required Nd:YAG capsulotomy due to a secondary cataract approximately 24 months after the procedure. Notably, no other significant intraoperative or postoperative complications were observed.

## 4. Discussion

To summarize, this is one of the longest long-term prospective single-center studies of glaucoma patients who have undergone phaco-ECP. At the final follow-up, the mean intraocular pressure (IOP) demonstrated a reduction exceedingly greater than a quarter. The qualified success rate was observed to be reasonable. The incidence of adverse events aligns with the findings reported in prior publications [15,18,19,20,21]. 

Lowering intraocular pressure (IOP) continues to be the principal and evidence-based strategy in the therapeutic management of glaucoma [22,23]. This can be achieved through various strategies, including pharmacological and surgical interventions aimed at reducing aqueous humor production and improving outflow. Within these approaches, inducing targeted damage to the non-pigmented epithelium of the ciliary body using methods like laser photocoagulation, cryotherapy, or ultrasound energy has been commonly practiced. Among these interventions, transscleral cyclophotocoagulation (TSCPC) stands out as the most prevalent and efficacious technique. The mechanism of action of TSCPC involves the destruction of the pigmented ciliary body epithelium, leading to indirect destruction of the non-pigmented cells and an increase in uveoscleral outflow [24,25]. This method is primarily used in severe cases of refractory glaucoma when prior pharmacological or surgical interventions, such as filtration or seton procedures, have proven ineffective [26]. 

Cyclodestruction techniques, including TSCPC, come with two major limitations in glaucoma treatment. Firstly, there is a lack of selectivity for the target tissue, which may lead to potential damage to adjacent structures. Secondly, predicting the therapeutic effect in relation to the applied dosage can be challenging. Additionally, TSCPC entails the risk of complications such as pain, conjunctival burn, scleral thinning, and uveitis [24,27,28,29]. While rare, more serious complications include hypotension, choroidal detachment, choroiditis, retinal detachment, and, extremely rarely, phthisis bulbi [7]. 

To address these challenges and enhance the safety profile of existing cyclophotocoagulation methods, two innovative approaches have emerged: micropulse cyclophotocoagulation and ultrasound cycloplasty, briefly mentioned in the introduction. In contrast to the transscleral methodology, endoscopic cyclophotocoagulation (ECP) enables the precise modulation of diode laser therapy, targeting the ciliary body by directly visualizing the tissue via endoscopy. The ability to directly observe the target tissue is the primary advantage of this method and has been proven to possess superior safety and selectivity over TSCPC [4,5,6]. Nonetheless, it is an invasive procedure typically indicated for patients with moderate glaucoma who are concurrently undergoing cataract surgery [14,15]. 

It is imperative to acknowledge that this procedure is not devoid of side effects. The most frequently observed complications include IOP spikes, heightened inflammation (in comparison to phacoemulsification without ECP), and the potential for intraocular lens dislocation [30,31]. In our study, corneal edema was noted in ten patients (26.3%), which spontaneously resolved over time without corneal decompensation or the need for corneal grafting. IOP spikes manifested in eight patients (21.1%), successfully managed with topical IOP-lowering medications, negating the necessity for further intervention. In one patient (2.6%), IOL dislocation (subluxation) was observed 36 months post-procedure, resulting in a decline in visual acuity. However, surgical intervention was not pursued, as the patient chose not to undergo the procedure. Hypotony was not observed in any of our patients. Macular edema occurred in three patients (7.9%) one month after the procedure and resolved within two months through pharmacotherapy, involving topical nonsteroidal anti-inflammatory drugs and oral carbonic anhydrase inhibitors. Epiretinal membranes were detected approximately 46 months after the initial procedure in four of our patients (10.5%), but surgical intervention was not required during the study follow-up due to the minimal progression of the condition. One patient (2.6%) developed a full-thickness macular hole 12 months after the initial procedure, necessitating subsequent vitrectomy surgery. Uveitis occurred in five of our patients (13.2%) approximately one month after the procedure. In four cases, it was minor and resolved after treatment with the topical steroid dexamethasone, without further complications. One patient developed fibrinous anterior uveitis with posterior synechiae formation, requiring intensive treatment with systemic prednisone therapy and subconjunctival steroid injections, which yielded positive results. No other significant intraoperative or postoperative complications were observed. The incidence of major complications in our study is comparable to the results published to date [15,18,19,20,21].

It is widely known that glaucoma is a progressive disease, and surgical interventions often need to be repeated periodically to maintain proper glaucoma control. Longer observation periods yield more data regarding the effectiveness of the procedure. Therefore, a crucial question in glaucoma management is how long a given procedure will suffice for maintaining proper glaucoma control. Moreover, there is a commonly held opinion that the ciliary body can regenerate after cyclodestruction, potentially leading to an increase in IOP and decreasing the long-term efficacy of the procedure, with the necessity of reperforming that procedure. In analyzing the limited literature on ciliary body regeneration, which is primarily based on animal studies, it remains challenging to definitively prove this opinion. A few studies have shown some epithelial regeneration in pars plicata [27] and the reperfusion of the ciliary processes [5,28], particularly in favor of ECP [5]. However, authors consistently conclude that the capillary network regeneration of the ciliary processes is mostly incomplete, and the regenerated epithelium is always disorganized, with a lack of a well-developed pigment epithelial layer, which may result in limited functionality concerning secretory function [27]. 

Many studies on ECP exhibit limited follow-up data, with our search identifying only one available study reporting 60-month outcomes and four studies presenting data with a 36months follow-up period. Other studies related to phaco-ECP have shorter follow-up durations. Oberfeld et al. [20] reported a 12% success rate at 60 months after phaco-ECP, with an 18% reduction in intraocular pressure (IOP) among the 9 patients who remained in the study out of the initial 78 enrolled. After 36 months, the same authors reported 27 patients still in the study, with a 25% reduction in IOP and a 34% success rate. Other studies with 36 months of follow-up showed a 25% reduction in IOP and a 40% success rate [18], a 26% reduction in IOP and a 45% success rate [19], or a 13% reduction in IOP with a 70% success rate [15,21]. Similarly, studies with a 24-month follow-up period reported IOP reductions ranging from 30% to 40% [32,33]. 

In our study, a decrease in IOP and the number of antiglaucoma medications was statistically significant at each follow-up time point. The mean IOP at the 60-month follow-up was reduced by 32.7%, with a qualified success rate of 40%. These results exceed those of the only available 5-year study conducted by Oberfeld et al. [20]. We attribute this difference to the extent of the ciliary process treatment, which in their study covered 90–360 degrees, whereas in our study it spanned 270–360 degrees. Other studies mentioned above, although with shorter follow-up durations, have reported higher success rates and IOP reductions, like our findings at the 36-month post-procedure mark, where the success rate of our study reached 60%. 

It is crucial to recognize the limitations associated with assessing intraocular pressure (IOP), particularly the presence of regression to the mean, an inherent challenge in non-randomized studies such as this one. To minimize the influence of IOP measurement variability, we conducted multiple assessments in accordance with the recommendations of the World Glaucoma Association [16]. While this approach helps mitigate the impact of regression to the mean, it remains a factor that warrants consideration when interpreting the results. Throughout the follow-up period, no significant changes in visual acuity were observed. While there was no statistically significant loss in the visual field (VF) at each follow-up time point within the study group, individual patient analyses revealed that a minority experienced substantial VF deterioration, typically necessitating re-treatment. Regarding different glaucoma subtypes, whether patients were treatment-naïve or had previously undergone surgical interventions, we did not identify any significant differences in outcomes following phaco-ECP.

It is noteworthy that phaco-ECP, due to its similar approach and low complication rate, should be compared to other glaucoma surgeries commonly performed in conjunction with phacoemulsification, such as an iStent injection, Hydrus Microstent implantation (HMS), or trabecular excision using a Kahook Dual Blade (KDB) as part of Microinvasive Glaucoma Surgery (MIGS). At the time of writing, publications directly comparing phaco-ECP to different combined procedures are limited. One publication that compared phaco-ECP with phaco-trabeculectomy showed similar IOP reductions at 24 months post operation [34]. With a shorter one-year follow-up period, Moghimi et al. [35] conducted a comparison between phaco-ECP, phaco-trabeculectomy, and phaco-viscocanalostomy, demonstrating the comparable effectiveness between the first two procedures and the superior effectiveness of the latter. Regarding trabeculectomy, it is noteworthy that common opinion and published clinical studies indicate a decreased success rate when it is combined with cataract surgery compared to when performed as a standalone procedure. This decrease is attributed to increased inflammation and bleb fibrosis [36,37]. In the aforementioned earlier study by Oberfeld et al. [20], notable for its extensive follow-up duration, a comparative analysis was conducted between phaco-ECP, phaco-KDB, and PEcK (combined phacoemulsification, ECP, and KDB). This study revealed similar results between the phaco-ECP and phaco-KDB groups but a greater reduction in the PEcK group [20]. Other published studies on ECP and MIGS compare combined phaco-ECP with or without other MIGS (KDB, iStent), presenting superior results in triple procedures [38,39]. Studies of MIGS procedures combined with cataract surgery in terms of extended observation, like our study with a 60-month follow-up, are limited and consist mainly of non-comparative studies or studies comparing phaco-MIGS to phaco alone. The Manchester iStent study even presented 7-year results, with a success rate of 46% of patients and a 20% IOP reduction after 60 months [40]. Arriola-Villalobos et al. reported outcomes from the same procedure, with a 16% decrease in IOP and a success rate of 40% among the 13 patients who completed 60 months of follow-up [41]. In contrast, while the two studies concerning iStent demonstrate a high success rate for that procedure, the HORIZON study, which focused on HMS procedures, presents quite the opposite results. After 5 years, there was a 6% reduction in IOP, and this was not statistically significant compared to phaco-alone procedures [42]. However, the authors found a reduced need for medication to achieve IOP control and a decreased need for incisional IOP-lowering procedures. The limited availability of studies with a 60-month follow-up period presents challenges in drawing definitive conclusions regarding the long-term effectiveness of these procedures and their comparison to phaco-ECP. More studies on MIGS, including phaco-ECP, with extended follow-up durations are undoubtedly necessary to provide a comprehensive assessment. This will help address the question of the most effective and safe treatment option for patients with uncontrolled glaucoma and concomitant cataract. It is important to note that comparing and analyzing the aforementioned studies is complicated due to variations in methodology, particularly in terms of defining a successful outcome.

The current study has several limitations, including its retrospective, noncomparative design and a moderate number of cases. Analyzing the results, these facts should be acknowledged and data validation is necessary from larger-scale studies. Nevertheless, it stands as one of the longest clinical studies and has the largest number of follow-up patients in the literature.

In conclusion, regarding ECP studies with a 60-month follow-up, only one study is available. Our study, conducted over the same follow-up period, demonstrates a reasonable success rate and IOP reduction, along with a favorable safety profile. By amalgamating this information from 60 months of follow-up data with our results after 36 months (where the success rate stands ranges from 40–70%) we can present a more comprehensive picture of the duration of ECP’s effects, which is satisfactory, in our opinion. These findings support its suitability as a viable option for patients with uncontrolled glaucoma and cataract. For this type of patient, when the cataract is operable, a combined procedure is recommended. In terms of patients with controlled glaucoma and operable cataract, despite this not being the primary focus of the study, and based on our experience, ECP is also a viable option to reduce the number of medications. However, in such cases, it is advisable to avoid inducing hypotony. Therefore, the number of drops and preoperative IOP should not be excessively low (low or mid-teens), considering that we are reducing the IOP by 30% with this method. Using this information, clinicians can determine the suitability and duration of a particular procedure in the treatment of glaucoma for individual patients. This is invaluable for the preservation of vision in glaucoma patients.

## 5. Conclusions

The phaco-ECP procedure is effective, well-tolerated, and safe for reducing the IOP in glaucoma patients with cataracts over a long-term follow-up. Randomized, larger-scale studies are required to validate the results obtained.

## Figures and Tables

**Table 1:** Demographic characteristics. Demographic data, type of glaucoma, and history of prior glaucoma surgeries of patients enrolled in the study.

Demographic Characteristics		
Age (years)		
Mean ± SD	78.03 ± 9.13	
Range	52.00–98.00	
	No. (%)	
Gender		
Female	24	(63.2)
Male	14	(36.8)
Type of glaucoma:		
Primary open-angle glaucoma	26	(68.4)
Secondary open-angle glaucoma		
Post-penetrating keratoplasty glaucoma	1	(2.6)
Exfoliative	3	(7.9)
Pigmentary	0	(0.0)
Uveitic glaucoma	1	(2.6)
Neovascular glaucoma	1	(2.6)
Other	3	(7.9)
Primary angle-closure glaucoma	3	(7.9)
Secondary angle-closure glaucoma	0	(0.0)
Aniridic glaucoma	0	(0.0)
Prior glaucoma surgeries:		
Trabeculectomy	4	
Deep sclerectomy	1	
Surgery (tube/stents)	0	
Transscleral cyclophotocoagulation	0	
Ultrasound Cyclo Plasty	0	

SD—standard deviation, MD—mean deviation.

**Table 2:** Glaucoma staging of patients enrolled in the study.

Glaucoma Staging		
	No. (%)	
Early glaucomatous loss MD ≤ 6 dB	16	(42.1)
Moderate glaucomatous loss 6 dB > MD ≤ 12 dB	13	(34.2)
Advanced glaucomatous loss MD > 12 dB	9	(23.7)

**Table 3:** Intraocular pressure and number of hypotensive medications taken after phaco-ECP—60-month follow-up.

	Mean IOP ± SD	*p*-Value	Number of Hypotensive Medications ± SD	*p*-Value	% IOP Reduction	No. Patients
Preop	22.6 ± 6.7		3.4 ± 0.9		-	38
1 day	18.4 ± 5.0	0.009	1.4 ± 1.4	<0.001	18.6	38
1 week	18.4 ± 7.3	0.007	1.6 ± 1.2	<0.001	18.5	38
1 month	16.4 ± 5.5	<0.001	1.9 ± 1.1	<0.001	27.3	38
3 months	16.8 ± 6.1	0.003	1.7 ± 0.9	<0.001	25.4	38
6 months	15.6 ± 3.7	<0.001	2.0 ± 1.2	<0.001	31.0	38
12 months	15.9 ± 3.9	<0.001	2.2 ± 1.1	<0.001	29.5	38
18 months	16.9 ± 5.0	<0.001	2.5 ± 1.2	<0.001	24.9	36
24 months	15.9 ± 2.9	<0.001	2.6 ± 1.2	0.001	29.4	35
30 months	16.6 ± 4.1	0.001	2.6 ± 1.3	0.005	26.4	33
36 months	15.6 ± 2.7	<0.001	2.6 ± 1.3	0.005	30.7	31
42 months	15.9 ± 4.5	0.006	2.3 ± 1.3	0.001	29.7	27
48 months	15.5 ± 3.8	0.001	2.1 ± 1.3	0.002	31.3	25
54 months	15.5 ± 2.8	<0.001	2.8 ± 0.9	0.013	31.4	23
60 months	15.2 ± 2.6	<0.001	2.7 ± 1.1	0.009	32.7	23

**Table 4:** Intraoperative and postoperative complications after phaco-ECP—60-month follow-up.

Complications		
	No. (%)	
Epithelial defects	0/38	(0.0)
Corneal edema	10/38	(26.3)
Corneal decompensation	0/38	(0.0)
IOP spike	8/38	(21.1)
IOL dislocation	1/38	(2.6)
Hypotony, choroid detachment	0/38	(0.0)
Macular edema	3/38	(7.9)
Macular hole	1/38	(2.6)
Epiretinal membrane	4/38	(10.5)
Retinal detachment	1/38	(2.6)
Phthisis bulbi	0/38	(0.0)
Uveitis	5/38	(13.2)

phaco-ECP—phaco-endocyclophotocoagulation, IOP—intraocular pressure, IOL—intraocular lens.

## Data Availability

The datasets generated during and/or analyzed during the current study are available from the corresponding author upon reasonable request.

## Abbreviations

IOP	Intraocular Pressure
MD	Mean Deviation
SS-OCT	Swept-Source Optical Coherence Tomography
ECP	Endocyclophotocoagulation
phaco-ECP	Phaco-endocyclophotocoagulation
TSCPC	Transscleral Cyclophotocoagulation
µCPC	Transscleral Microcyclophotocoagulation
UCP	Ultrasound Ciliary Plasty
KDB	Kahook Dual Blade
MIGS	Microinvasive Glaucoma Surgery
HMS	Hydrus Microstent implantation
